# Potential Role of Adjuvant Lenvatinib in Improving Disease-Free Survival for Patients With High-Risk Hepatitis B Virus-Related Hepatocellular Carcinoma Following Liver Transplantation: A Retrospective, Case Control Study

**DOI:** 10.3389/fonc.2020.562103

**Published:** 2020-12-07

**Authors:** Bing Han, Han Ding, Shuai Zhao, Yichi Zhang, Jian Wang, Yue Zhang, Jinyang Gu

**Affiliations:** ^1^Department of Transplantation, Xinhua Hospital Affiliated to Shanghai Jiao Tong University School of Medicine, Shanghai, China; ^2^Department of General Surgery, Xinhua Hospital Affiliated to Shanghai Jiao Tong University School of Medicine, Shanghai, China; ^3^Department of Bioinformatics and Biostatistics, School of Life Sciences and Biotechnology, Shanghai Jiao Tong University, Shanghai, China

**Keywords:** hepatocellular carcinoma, liver transplantation, adjuvant therapy, lenvatinib, recurrence

## Abstract

**Background and Aim:**

Although liver transplantation (LT) is one of the most effective treatments for the patients with hepatocellular carcinoma (HCC), the high-risk patients suffer from a high ratio of tumor recurrence after LT. Lenvatinib, as a novel targeted drug, has shown an excellent effect in the treatment of advanced HCC, but there is no study on its effect in preventing HCC recurrence in the patients undergoing transplantation. Therefore, this study was designed to evaluate the role of adjuvant lenvatinib in preventing recurrence of high-risk LT recipients with HBV-related HCC.

**Methods:**

We retrospectively analyzed 23 high-risk patients consisting of lenvatinib group (n=14) and control group (n=9) with HBV-related HCC who underwent LT in our center. Disease-free survival (DFS) and HCC recurrence of the two groups were compared. The adverse events (AEs) and drug tolerance of lenvatinib were evaluated.

**Results:**

The median DFS in lenvatinib group was 291 (95%CI 204–516) days, significantly longer than 182 (95%CI 56–537) days in control group (P=0.04). Three patients in lenvatinib group (21.4%) and five patients in control group (55.6%) had short-term HCC recurrence (P=0.11). All patients in lenvatinib group could tolerate oral lenvatinib for at least three cycles except six cases (42.9%) of dose reduction and 1 case of interruption (14.3%). Thirteen patients (92.9%) taking lenvatinib experienced AEs. The most common AEs were hypertension (64.3%) and proteinuria (42.9%), and the most serious AEs were Grade 3 for 4 cases (28.5%) according to common terminology criteria for adverse events (CTCAE) version 5.0. Additionally, no influence of lenvatinib on the dosage and blood concentration of FK506 was observed.

**Conclusions:**

Adjuvant lenvatinib had a potential benefit on prolonging the DFS and reducing the recurrence of high-risk HBV-related HCC patients following liver transplantation with an acceptable drug safety and patient tolerance.

## Introduction

Hepatocellular carcinoma (HCC) accounts for more than 80% of all primary liver cancer, which is the sixth most commonly diagnosed cancer and the fourth leading cause of cancer-related death worldwide in 2018, with about 841,000 new cases and 782,000 deaths annually (1). In China, it is the third most common malignancy with a 5-year survival rate of 12.1%, the second lowest in all invasive cancers (2). Since liver transplantation (LT) was introduced as an unprecedented and potentially curative operation for unresectable hepatocellular carcinoma (HCC), the number of LT candidates with HCC as primary indication has grown continuously worldwide, representing up to 50% of the indications in most transplant centers (3).

Although LT is the only treatment that offers the real chance to eradicate both HCC and the underlying liver cirrhosis, recurrence of HCC remains unavoidable and is one of the main causes of death after LT (4). Milan criteria (MC) has been the most widely used indication criteria for LT candidates with HCC, and 5-year overall survival (OS) rate and disease-free survival (DFS) rate of patients meeting MC after transplantation is about 70% and 60% (5, 6). Because Milan criteria was so strict to deprives the transplantation opportunities of some HCC patients who might have benefited from LT, some other criteria, such as UCSF criteria, Pittsburgh criteria, Tokyo criteria, and Hangzhou criteria, has been proposed and expanded the indication of LT for HCC in varying degrees (7–9). Unfortunately, 5-year oval survival rate and recurrence-free survival rate falls to approximately 50% and 35% respectively in patients with advanced HCC who receive liver transplants under extended criteria and who can be considered to be at high-risk of HCC recurrence after transplantation (6, 10, 11). A strategy for prolonging both DFS and OS in such high-risk patients with HCC is a challenging but critical issue. In order to reduce HCC recurrence after LT, some randomized studies attempting to use chemotherapeutic drugs as adjuvant therapy after LT has suggested no obvious benefit for HCC patients (12). In last decade, several studies in which sorafenib was taken as adjuvant therapy following LT of high-risk HCC candidates just demonstrated a potential effect on reducing recurrence (5, 13, 14). Therefore, there is still no clear consensus on the adjuvant therapy after LT for preventing HCC recurrence.

Recently, lenvatinib, a novel molecule-targeted drug behaving as an inhibitor of vascular endothelial growth factor (VEGF) receptors, fibroblast growth factor (FGF) receptors, platelet-derived growth factor (PDFG) receptor alpha and KIT and RET proto-oncogenes, completed the phase II clinical trial and a randomized phase 3 non-inferiority trial (REFLECT trial) in which lenvatinib showed non-inferior to sorafenib in overall survival, as well as statistically significant improvement in progression-free survival, time to progression, and objective response rate (ORR) with safety (15–17). Interestingly, subgroup analysis revealed that the OS of HBV-related HCC patients treated with lenvatinib was significantly longer than that of the sorafenib group, which suggested that lenvatinib may be more effective for hepatitis B-related HCC. Hereupon, lenvatinib was recommended as a first-line drug for unresectable HCC in several clinical practice guidelines, including National Comprehensive Cancer Network (NCCN), European Society for Medical Oncology (ESMO), American Association for the Study of Liver Diseases (AASLD), European Association for the Study of the Liver (EASL) and Chinese Society of Clinical Oncology (CSCO) (18–20). To date, there were few reports on application of lenvatinib in LT recipients with HCC, especially no report of using LEN as adjuvant therapy for preventing HCC recurrence after LT. Therefore, a retrospective case control study (NCT04415567) was conducted by reviewing 23 high-risk HCC patients after LT in our department, 14 of whom taking lenvatinib as adjuvant therapy after LT, and aimed to evaluate the safety and effect of adjuvant therapy using lenvatinib in these patients.

## Patients and Methods

### Patients

We retrospectively reviewed 23 Chinese HCC patients with HBV infection, who underwent LT in our hospital from June 2018 to December 2019. All donor grafts were allocated by the China Organ Transplant Response System. All these patients were diagnosed by histology and were defined as “high-risk” for recurrence according to the following criteria: (1) beyond Milan criteria confirmed either by radiology before LT or by pathology after LT, (2) tumor with intrahepatic vascular invasion, (3) Alpha-fetoprotein (AFP)≥400 ng/L before LT, (4) presence of microvascular invasion (MVI), (5) tumor with histological poor differentiation according to Edmondson-Steiner classification system (21), (6) multiple satellite lesions around the largest tumors detected either by radiology before LT or by histology after LT, (7) tumor penetrating hepatic capsule, (8) recurrent HCC after resection.

Patients were divided into lenvatinib group and control group according to their willingness to take lenvatinib as adjuvant therapy after LT. Of the 23 patients, 14 patients in lenvatinib group began to take lenvatinib about a month after LT except for routine treatment, while the other nine patients in control group received routine treatment and follow-up after transplantation. Clinical data and demographic characteristics was obtained, including age, sex, underlying liver disease, presence of pre-transplant treatments, LT-related information and tumor pathology. This study was conducted according to the 1975 Declaration of Helsinki and approved by Ethics Committee of Xin Hua Hospital Affiliated to Shanghai Jiao Tong University School of Medicine (No. XHEC-D-2020-068). All patients enrolled in this study provided informed consent.

### Usage of Lenvatinib and Immunosuppressants

The patients in lenvatinib group received oral lenvatinib (Eisai, Japan) 12 mg/day (for bodyweight (BW) ≥60 kg) or 8 mg/day (for BW <60 kg) in 28-day cycles until HCC recurrence or serious adverse events (SAEs) or voluntary withdrawal. Dose interruptions followed by reductions for lenvatinib-related toxicities (to 8 mg and 4 mg/day, or 4 mg every other day) were permitted. All 14 patients took lenvatinib for more than three cycles.

The induction immunosuppression strategies for all patients enrolled in the study involved IV infusion of 20 mg of basiliximab within 2 h prior to operation and a second dose 4 days later, oral tacrolimus started on the fourth day after LT at a dose of 0.04 mg/kg (BW) and adjusted according to its plasma concentration, taking mycophenolate mofetil (MMF) from the next day after surgery at a dose of 500 mg/kg (BW), and rapid withdrawal of glucocorticoids with the initial dose of 500 mg. Maintenance immunosuppression which was started about one month after LT included sirolimus (4 mg/M2 per day) plus oral tacrolimus with the plasma concentration maintained at 5–8 ng/ml.

### Following Up and Clinical Assessment

All patients were followed up monthly within six months after LT and every three months within two years. During each follow-up, complete blood count (CBC), urinalysis, serum AFP level, liver and kidney function test, and blood concentration of FK506 were recorded. Chest and abdominal computed tomography was implemented at 3 months, 6 months, 12 months, and annually thereafter. Other radiological examinations such as radionuclide bone scan, magnetic resonance imaging (MRI) and positron emission tomography (PET) were obtained when local recurrence or distant metastasis was suspected. HCC recurrence was diagnosed by the definite tumor lesions found in radiology.

The DFS was defined as the period between the day of LT and the day of HCC recurrence and metastasis confirmed by imaging, while the OS was defined as the duration from LT to death of patients for any reason or to end of follow-up. Common terminology criteria for adverse events version 5.0 (CTCAE V5.0) was used to assess the AE during oral administration of lenvatinib. The FK506 dosage and blood concentration of each patients in the first six months after liver LT was recorded for evaluate the influence of lenvatinib on the immunosuppressive therapy.

### Statistical Analysis

Mean and standard deviations were used for descriptive statistics. The patient characteristics in each group were compared by one-way ANOVA and chi-square tests. Repeated measures analysis of variances was used for comparing the difference of FK506 dosage and blood concentration between two groups. The OS and DFS were statistically analyzed by the Kaplan-Meier method. Statistical significance was set at P<0.05. All statistical analyses were performed using SPSS software Version 10.0.

## Results

### Demographic Characteristics and Clinical Data

The comparison of demographic characteristics and clinical data between two groups was shown in **Table 1**, in which we concluded that there was no significant difference in baseline data between the two groups. The median follow up was 468 days (95%CI 258–616) in lenvatinib group and 445 days (95%CI 180–673) in control group (χ2 = 0.977, P=0.324). In lenvatinib group, all patients took for more than 3 months, with a shortest medication time of 90 days and a longest time of 512 days. Two patients withdrew the administration of lenvatinib due to HCC recurrence, three patients stop taking the drug for AEs of grade 3, and the other 3 patients stop lenvatinib treatment for cost. Five patients have been taking lenvatinib orally without recurrence.

**Table 1:** Comparison of demographic characteristics and clinical data between two groups.

	Lenvatinib	Control	χ^2^ value	*P* value
Sex (male/female)	14/0	9/0	—	—
Age (Mean ± SD)	51 ± 11.8	50 ± 17.3	0.07	0.80
Comorbidity (%)	3(21.4%)	1(11.1%)	0.41	0.48
Pre-LT treatment				
TACE (%)	5(35.7%)	5(55.6%)	0.88	0.1
RFA	0	1(11.1%)	1.63	0.39
TIPS	1(7.1%)	0	0.67	0.61
Sorafenib	1(7.1%)	0	0.67	0.61
Blood group (A/B/O/AB)	4/5/4/1	2/3/3/1	0.23	0.97
Donor-recipient ABO Compatibility (identical/compatible/in compatible)	12/2/0	7/2/0	0.24	0.52
Pre-LT HBV-DNA (positive/negative)	5/9	3/6	0.01	0.63
Surgical complication (%)	9(64.3%)	3(33.3%)	2.10	0.15
Clavien-Dindo Grade (0/I/II/III/IV/V)	5/0/0/9/0/0	6/1/0/2/0/0	4.68	0.10
Histological Poor Differentiation (%)	9(64.3%)	4(44.4%)	0.88	0.31
R0 resection (%)	11(78.6%)	9(100%)	2.22	0.21
Underlying liver cirrhosis (%)	13(92.9%)	8(88.9%)	0.11	0.64
Child-Pugh Classification (A/B/C)	11/3/0	5/3/1	2.27	0.32
MELD score (Mean ± SD)	10 ± 2.5	11 ± 3.5	0.23	0.64
Beyond Milan Criteria (%)	11(78.6%)	5(55.6%)	1.37	0.24
Tumor Number>3(%)	8(57.1%)	6(66.7%)	0.70	0.36
largest tumor diameter>5cm (%)	8(57.1%)	5(55.6%)	0.01	0.64
PVTT (%)	4(40.0%)	3(33.3%)	0.06	0.58
MVI (%)*	3(30%)	0	2.22	0.21
AFP≥400 ng/L (%)	5(35.7%)	6(66.7%)	2.10	0.15
Multiple Satellite Lesions (%)	7(50.0%)	4(44.4%)	0.07	0.57
Hepatic Capsule Invasion (%)	5(35.7%)	3(33.3%)	0.01	0.63
Post-resection Recurrence (%)	3(21.4%)	2(22.2%)	0.00	0.67

Comorbidity: including hypertension, diabetes, and history of cerebral infarction, TACE, transarterial chemoembolization; RFA, radiofrequency ablation; TIPS, transjugular intrahepatic portosystemic shunt; PVT, portal vein tumor thrombus; MVI, microvascular invasion. Definition of histological Poor Differentiation: lower than moderate differentiation, *MVI was not detected for the patients with PVTT.

### Effect of Lenvatinib

**Figure 1:**
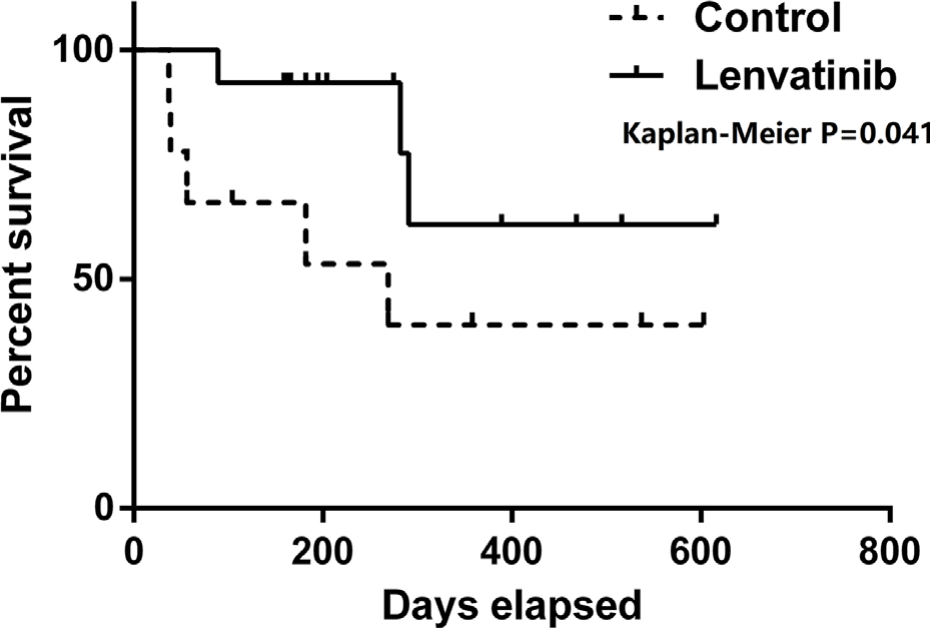
Disease-free survival (DFS) of two groups. Patients in lenvatinib group had better DFS than those in control group (*P* = 0.04).

**Figure 2:**
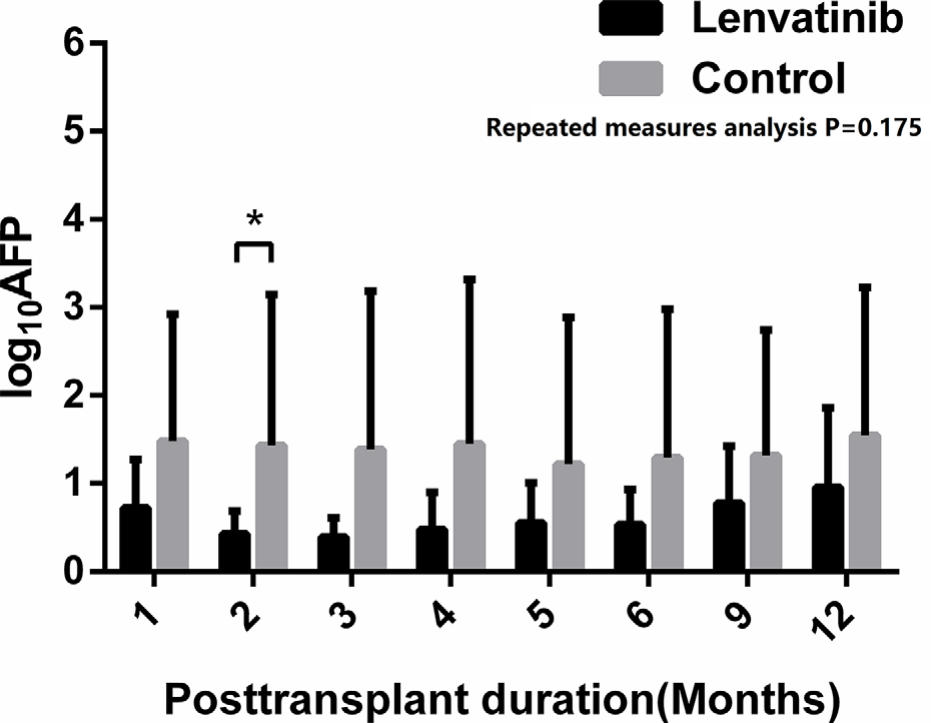
Alpha-fetoprotein (AFP) level of the patients after liver transplantation. No significant difference between two groups was found by repeated measures analysis (P=0.175), but the value of log_10_AFP in lenvatinib group was significantly lower than that in control group in the second month after liver transplantation (LT) (P=0.04) *: P < 0.05.

The efficacy of lenvatinib was shown in **Table 2**. **Figure 1** depicts the DFS of our cohorts. All patients are alive at present till the end of the study, so the median OS of patients in our cohorts was 468 days (95%CI 258–616) in lenvatinib group and 445 days (95% CI 180–673) in control group, the same with follow-up (χ2 = 0.977, P=0.324). The median DFS in lenvatinib group was 291 (95% CI 204–516) days, compared with 182 (95%CI 56–537) days in control group. There was significant difference between two groups by the Kaplan-Meier method (χ2 = 4.208, P=0.041). Three patients in lenvatinib group (21.4%) and five patients in control group (55.6%) had short-term HCC recurrence, but no significant difference was found between two groups (χ2 = 2.813, P=0.11). In both groups, pulmonary recurrence was the most common site of recurrence, which involved in 2 cases (14.3%) in lenvatinib group and 3 cases (33.3%) in control group. One patient in lenvatinib group had multiple recurrences in lung and left adrenal gland, while one patient in control group suffered from multiple recurrence in lung and sacral bone. By repeated measures analysis, no significant difference was observed in the AFP level during the first year after LT between two groups (F=1.996, P=0.175) (**Figure 2**).

**Table 2:** Efficacy measures.

	Lenvatinib	Control	χ2 value	P value
DFS (95%CI)	291(204–516)	182(46–447)	4.16	0.04
OS (95%CI)	468(258–616)	445(180–638)	0.97	0.32
Recurrence (%)	3(21.4%)	5(55.6%)	2.81	0.11
Lung	2(14.3%)	3(21.4%)	1.17	0.28
Adrenal gland (%)	1(7.1%)	1(7.1%)	0.11	0.64
Intrahepatic (%)	1(7.1%)	0	0.67	0.61
Bone (%)	0	1(7.1%)	1.63	0.39

### Adverse Events of Lenvatinib

All patients in lenvatinib group could tolerate the oral lenvatinib for at least three cycles, but six of them (42.9%) underwent dose reduction due to AEs of Grade 2 and another patient (14.3%) experienced one-week drug interruption followed by dose reduction because of nasal bleeding. However, AEs of grade 3 led to lenvatinib withdraw in three patients (21.4%). **Table 3** lists the adverse events during lenvatinib administration and the corresponding CTCAE grade. Total incidence of AEs was 92.9% (13/14). The most common AEs were hypertension (64.3%) and proteinuria (42.9%). No fatal AE happened and the most serious AEs were four cases of CTCAE Grade 3, including hypertension, drug-induced liver injury (DILI), fatigue and nasal bleeding.

**Table 3:** Adverse events and their corresponding common terminology criteria for adverse events (CTCAE) grade.

AEs	Grade1	Grade 2	Grade 3	Grade 4	Grade 5	Total
Proteinuria	2(14.3%)	4(28.6%)	0	0	0	6(42.9%)
Hypertension	3(21.4%)	5(35.7%)	1(7.1%)	0	0	9(64.3%)
PPES	4(28.6%)	1(7.1%)	0	0	0	5(35.7%)
Diarrhea	4(28.6%)	1(7.1%)	0	0	0	5(35.7%)
Decreased appetite	1(7.1%)	2(14.3%)	0	0	0	3(21.4%)
DILI	0	1(7.1%)	1(7.1%)	0	0	2(14.3%)
Arthralgia	1(7.1%)	2(14.3%)	0	0	0	3(21.4%)
Fatigue	1(7.1%)	0	1(7.1%)	0	0	2(14.3%)
Dysphonia	1(7.1%)	1(7.1%)	0	0	0	2(14.3%)
Alopecia	1(7.1%)	0	0	0	0	1(7.1%)
Gingival bleeding	1(7.1%)	0	0	0	0	1(7.1%)
Nasal bleeding	0	0	1(7.1%)	0	0	1(7.1%)
Chest tightness	2(14.3%)	0	0	0	0	2(14.3%)

PPES, palmar-plantar erythrodysesthesia syndrome; DILI, drug-induced liver injury.

### Influence of Lenvatinib on Immunosuppressant

**Figure 3:**
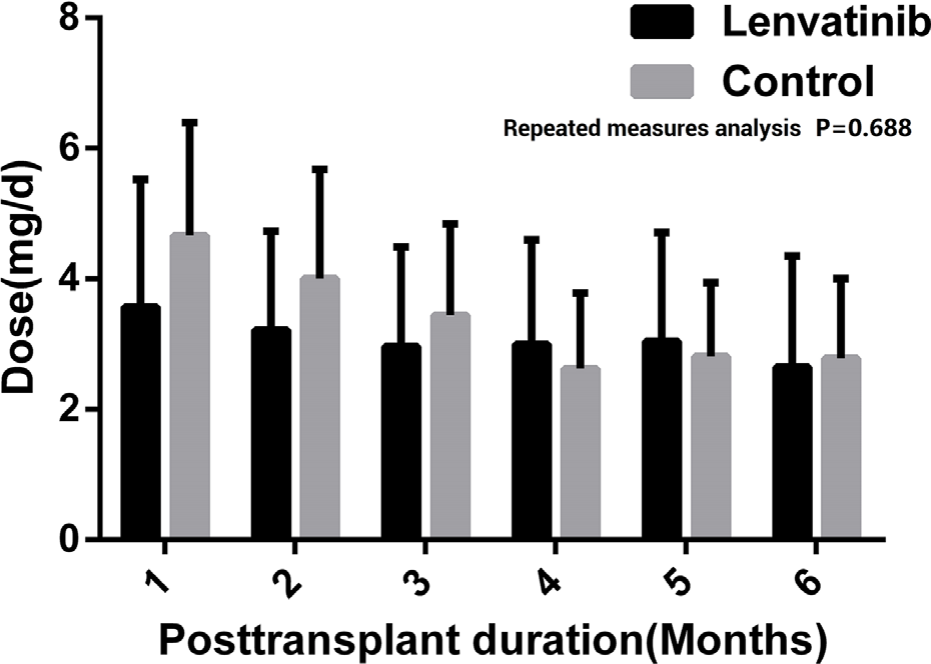
Using dose and blood concentration of FK506 during the first 6 months after liver transplantation (LT) **(A)**. Dose of FK506 **(B)**. Blood concentration of FK506.

There was no significant difference in dosage of FK506 during the first six months after LT between two groups (F=0.167, P=0.688), as well as the blood concentration (F=2.439, P=0.139). **Figure 3** shows the dosage and blood concentration of FK506 in both groups, with no difference in monthly results.

## Discussion

HCC is one of the most common cancers worldwide with the incidence rising over the last 20 years (22). Since the Milan criteria has been widely accepted, LT has gradually become a superior way to LR or other locoregional treatments for HCC patients who met the criteria, because it enabled the widest possible resection margins and completely removes the diseased liver at risk of developing HCC (23). However, the debate regarding the feasibility of transplantation for patients beyond the Milan criteria has not been resolved thus far, since the recurrence rate was reported rising in varying degrees (6, 10, 11). However, LT remains the only possible curative treatment for these patients who was considered to be at high risk of recurrence after transplantation.

In addition, some other factors have been identified as high risk factors for HCC recurrence after transplantation. It was widely proved that macrovascular and microvascular invasion, high AFP level before LT, as well as poor tumor differentiation are the most important factors affecting HCC recurrence (6, 24). AFP and tumor differentiation as an important index were involved in Hangzhou standard, as well as the latest AFP model and Up-to-Seven criteria Metroticket V2.0 (9, 25). Besides, some other researchers proposed that MVI is also one of the important factors influencing HCC recurrence after LT through Cox regression analysis (4, 26). Li et al. demonstrated in their study that exceeding Milan criteria, macrovascular invasion, liver capsule invasion and satellite lesions were significant different between the patients with and without HCC recurrence after LT by univariate analysis, implying they were high risk factors for HCC recurrence (27). Based on these reports and the experience in our center, we adopted the above-mentioned high-risk factors for recurrence in our study.

Before lenvatinib, sorafenib was the first and only one molecule-targeted drug approved for HCC treatment, so several studies have been carried out to evaluate the effect of sorafenib on preventing from the HCC recurrence of patients after LT. A retrospective study from Satapathy and his colleagues showed that preemptive treatment with sorafenib in OLT recipients with high-risk features in explant did not improve HCC recurrence-free or overall survival (28). Shetty et al. demonstrated decreased overall rate of HCC recurrence rate and prolonged 1-year disease free survival of patients by adjuvant sorafenib after LT, concluding that adjuvant use of sorafenib could decreases risk of HCC recurrence in high-risk LT recipients (29). In a multicenter phase I trial of adjuvant sorafenib in 14 LT recipients with high-risk HCC, one patient (7.1%) died and four (28.5%) recurred over a median follow-up of 953 days, implying a potentially promising effect of post-transplant sorafenib on recurrence-free survival (5). Another study demonstrated the safety and potential benefit of sorafenib in reducing the incidence of HCC recurrence and in extending disease-free and overall survival for high-risk liver transplant recipients (13). In a case control study, the disease-free survival at 6 months, 12 months and 18 months and the overall survival rate at 24 months for patients with adjuvant sorafenib were increased significantly (14). Accordingly, there is no certain conclusion about the adjuvant use of sorafenib in patients with high-risk HCC after transplantation.

Lenvatinib is a novel oral multi-kinase inhibitor that targets VEGF receptors 1–3, FGF receptors 1–4, PDGF receptor α, RET, and KIT. In 2018, lenvatinib was proved to be non-inferior to sorafenib in overall survival in untreated advanced HCC in the randomized phase III trial, in which the patients with HBV-related HCC in lenvatinib group had a superior PFS and OS to the patients in sorafenib group (15). Therefore, we conducted this retrospective study. As far as we know, this is the first report to describe the potential role of lenvatinib as adjuvant therapy in reducing HCC recurrence after liver transplant for high-risk patients.

Since lenvatinib was just approved for HCC treatment in March, 2018 in Japan, which is the first country in the world approving lenvatinib for HCC treatment and a number of studies revealed that postoperative recurrence of HCC often occurred in early stage after LT (14), this study involved twenty-three high-risk HCC patients who underwent LT in recent two years. Although lenvatinib only being SFDA-approved for advanced HCC in September 2018, before that, two patients who took lenvatinib purchased the drugs from abroad themselves and one of them relapsed after administration of lenvatinib for 7 months. Within our cohorts, there was a significant longer DFS in lenvatinib group, which inferred a certain benefit of adjuvant lenvatinib on prolonging the DFS of HBV-related high-risk HCC patients after LT. Although no significant statistical difference was observed in recurrence rate between two groups, this study provides initial but important evidence that adjuvant lenvatinib could be effective for decreasing HCC recurrence of high-risk patients following transplantation. We found extrahepatic recurrences, especially in the lung, were the more common in early postoperative days, which was observed in the former study (27). In lenvatinib group, one of three recurrent patients who experienced intrahepatic multiple recurrence received TACE and replacement of anlotinib, another target drug. Another patient with lung metastasis had pulmonary surgery and anlotinib instead as well. The third patient suffering from multiple recurrences continued to oral lenvatinib without dose change and lived for 14 months after recurrence. In control group, four recurrent patients started oral lenvatinib when diagnosis of HCC recurrence except for one patient who was found HCC metastasis in his left adrenal gland underwent resection of left adrenal gland. All patients in both groups survival so far, resulting in no significant difference in OS. To sum up the efficacy, adjuvant lenvatinib had a potential role in increasing the DFS and decreasing the recurrence rate for high-risk HBV-related HCC patients following LT. Moreover, no obvious effect of adjuvant lenvatinib on serum AFP level in the patients with high-risk HCC after liver transplantation was found in the study.

In terms of lenvatinib safety, total incidence of AEs in our study was 92.9% (13/14), similar with the previous trials, as all 46 patients experienced at least one AE in phase II trial and 94%of subjects had varying degrees of AEs (15, 17). However, in our cohorts, the incidence of Grade 3 AEs was 28.5% (4/14) with no AEs beyond Grade 3, obviously lower than the rate of 48% in phase II trial and 57% in phase III trial (15, 17). This may be resulted from the small number of cases in this study, the better basic physical condition of the patients who could went through LT, and the elimination of the patients’ underlying liver diseases by LT. Besides, in both of this study and previous trials, the most common AE was hypertension with similar incidence (64.3% vs 76.1% vs 42%). In our cohorts, treatment-related AEs led to lenvatinib drug interruption in one patient (7.1%), dose reduction in 6 patients (42.9%), and drug withdrawal in 3 patients (21.4%), lower than those in phase III trial. It inferred that patients after LT might behave a superior tolerance to lenvatinib.

As a special group, the interaction between any additional medication and the immunosuppressants should be considered for the LT recipients, so the dose and blood concentration change of FK506 was detected in the study to evaluate whether lenvatinib would influence the FK506 usage. Since the patients in lenvatinib group began lenvatinib administration about 1 month after surgery, we compared the dose and blood concentration of FK506 during the first six months after LT between the two groups, founding no significant difference. The results suggested no obvious influence of lenvatinib on the use of CNI immunosuppressants of the LT recipients.

In conclusion, this preliminary study demonstrates a potential benefit of adjuvant lenvatinib on prolonging the disease-free survival and reducing the recurrence of high-risk patients with HBV-related HCC following liver transplantation with an acceptable drug safety and patient tolerance. Limitations of this study include its retrospective analysis, and only one center experience, small number of patients involved as a result of strict selection criteria. Therefore, a prospective and randomized study with large sample should be encouraged.

## Data Availability Statement

The raw data supporting the conclusions of this article will be made available by the authors, without undue reservation.

## Ethics Statement

This study was approved by the Ethics Committee of Xin Hua Hospital Affiliated to Shanghai Jiao Tong University School of Medicine. All patients enrolled in this study signed informed consent.

## Author Contributions

BH and JG contributed to the conception and design of the study. BH wrote the manuscript and JG revised it. YuZ, HD, and SZ contributed to data analysis. YiZ and JW contributed to data collection. All authors contributed to the article and approved the submitted version.

## Funding

This study was supported by the National Natural Science Foundation of China (81772507, 82072645, 81300338), Foundation for Shanghai Jiao Tong University for SMC morning Star Youth Scholars Program, Shanghai Shenkang Program (SHDC2020CR3005A), Program of Medical Engineering Cross Research Fund of Shanghai Jiao Tong University (YG2017MS50).

## Conflict of Interest

The authors declare that the research was conducted in the absence of any commercial or financial relationships that could be construed as a potential conflict of interest.
